# Comparison of somatostatin receptor expression in patients with neuroendocrine tumours with and without somatostatin analogue treatment imaged with [^18^F]SiTATE

**DOI:** 10.3389/fonc.2023.992316

**Published:** 2023-01-30

**Authors:** Ralf S. Eschbach, Markus Hofmann, Lukas Späth, Gabriel T. Sheikh, Astrid Delker, Simon Lindner, Klaus Jurkschat, Carmen Wängler, Björn Wängler, Ralf Schirrmacher, Reinhold Tiling, Matthias Brendel, Vera Wenter, Franziska J. Dekorsy, Mathias J. Zacherl, Andrei Todica, Harun Ilhan, Freba Grawe, Clemens C. Cyran, Marcus Unterrainer, Johannes Rübenthaler, Thomas Knösel, Tanja Paul, Stefan Boeck, Christoph Benedikt Westphalen, Christine Spitzweg, Christoph J. Auernhammer, Peter Bartenstein, Lena M. Unterrainer, Leonie Beyer

**Affiliations:** ^1^ Department of Nuclear Medicine, University Hospital, LMU Munich, Munich, Germany; ^2^ Fakultät für Chemie und Chemische Biologie, Technische Universität Dortmund, Dortmund, Germany; ^3^ Biomedical Chemistry, Clinic of Radiology and Nuclear Medicine, Medical Faculty Mannheim of Heidelberg University, Mannheim, Germany; ^4^ Medical Faculty Mannheim of Heidelberg University, Molecular Imaging and Radiochemistry, Clinic of Radiology and Nuclear Medicine, Mannheim, Germany; ^5^ Department of Oncology, Division of Oncological Imaging, University of Alberta, Edmonton, AB, Canada; ^6^ ENETS Centre of Excellence, Interdisciplinary Center of Neuroendocrine Tumours of the GastroEnteroPancreatic System at the University Hospital of Munich (GEPNET-KUM), University Hospital of Munich, Munich, Germany; ^7^ Department of Radiology, University Hospital, LMU Munich, Munich, Germany; ^8^ Institute of Pathology, LMU, Munich, Germany; ^9^ Department of Internal Medicine 3, University Hospital, Munich, Germany; ^10^ Department of Internal Medicine 4, University Hospital, LMU Munich, Munich, Germany

**Keywords:** NET, PET/CT, [^18^F]SiTATE, somatostatin analogues, somatostatin receptor, molecular imaging

## Abstract

**Purpose:**

Somatostatin analogues (SSA) are frequently used in the treatment of neuroendocrine tumours. Recently, [^18^F]SiTATE entered the field of somatostatin receptor (SSR) positron emission tomography (PET)/computed tomography (CT) imaging. The purpose of this study was to compare the SSR-expression of differentiated gastroentero-pancreatic neuroendocrine tumours (GEP-NET) measured by [18F]SiTATE-PET/CT in patients with and without previous treatment with long-acting SSAs to evaluate if SSA treatment needs to be paused prior to [18F]SiTATE-PET/CT.

**Methods:**

77 patients were examined with standardised [18F]SiTATE-PET/CT within clinical routine: 40 patients with long-acting SSAs up to 28 days prior to PET/CT examination and 37 patients without pre-treatment with SSAs. Maximum and mean standardized uptake values (SUVmax and SUVmean) of tumours and metastases (liver, lymphnode, mesenteric/peritoneal and bones) as well as representative background tissues (liver, spleen, adrenal gland, blood pool, small intestine, lung, bone) were measured, SUV ratios (SUVR) were calculated between tumours/metastases and liver, likewise between tumours/metastases and corresponding specific background, and compared between the two groups.

**Results:**

SUVmean of liver (5.4 ± 1.5 vs. 6.8 ± 1.8) and spleen (17.5 ± 6.8 vs. 36.7 ± 10.3) were significantly lower (p < 0.001) and SUVmean of blood pool (1.7 ± 0.6 vs. 1.3 ± 0.3) was significantly higher (p < 0.001) in patients with SSA pre-treatment compared to patients without. No significant differences between tumour-to-liver and specific tumour-to-background SUVRs were observed between both groups (all p > 0.05).

**Conclusion:**

In patients previously treated with SSAs, a significantly lower SSR expression ([18F]SiTATE uptake) in normal liver and spleen tissue was observed, as previously reported for 68Ga-labelled SSAs, without significant reduction of tumour-to-background contrast. Therefore, there is no evidence that SSA treatment needs to be paused prior to [18F]SiTATE-PET/CT.

## 1 Introduction

Overexpression of somatostatin receptors (SSRs) is highly relevant for both diagnostics and therapeutic options in well-differentiated neuroendocrine tumours (NET) (1, 2). According to the German and European consensus guidelines, treatment with somatostatin analogues (SSAs) is the first line treatment for proliferation control in all well-differentiated metastatic/non-resectable NET of the Gastro-Entero-Pancreatic System (GEP-NET) (3–5). Diagnostically, ^68^Ga-labeled SSAs are recommended for staging, re-staging and therapy monitoring (6, 7). Therapeutically, ^177^Lu-/^90^Y-labeled SSAs are used in later-stage disease in metastatic/non-resectable GEP-NETs (4, 5).

As both therapeutic and diagnostic SSAs bind to SSRs, medication with SSA could potentially reduce the specific binding of the SSA radiotracer in combined positron-emission-tomography/computed tomography (PET/CT) imaging. After treatment with SSA octreotide, *in vitro* studies indicated internalisation of SSR subtype 2 receptors (8–11). On the contrary, one study suggested upregulation of SSR expression after incubation of pituitary cells in culture with a SSA (12).

For imaging, a former study revealed an improved visualisation of carcinoid liver metastases by ^111^In-pentetreotide scintigraphy after treatment with cold SSA (13). The uptake of ^68^Ga-labeled SSAs DOTATATE and DOTATOC in PET/CT imaging was found to be only reduced in the normal organs but not in tumour tissue after SSA medication, leading to an even higher tumour-to-background contrast (14, 15). To rule out a potentially reduced binding and impaired therapeutic effect of radioactive SSA for peptide receptor radionuclide therapy (PRRT), medication with long-lasting SSAs needs to be paused at least 30 days and medication with short-acting SSAs for at least 24 hours prior to PRRT (16).

Currently, the first ^18^F-labelled SSA radioligand, [^18^F]SiTATE has been introduced as an alternative to ^68^Ga-labeled SSAs for NET SSR-PET imaging with comparable radiation exposure and promising tumour-to-background contrast (17–20). The aim of this study was to investigate the influence of SSA medication prior to [^18^F]SiTATE PET/CT regarding normal-tissue and tumour uptake of the radiotracer when compared to former ^68^Ga-labeled SSAs to validate its clinical potential.

## 2 Materials and methods

### 2.1 Patient enrolment

All patients were referred for imaging by their treating endocrinologists and/-or oncologists between March 2019 and April 2021 and gave written informed consent to undergo [^18^F]SiTATE-PET/CT following the regulations of the German Pharmaceuticals Act. In principle, all patients with a NET were included, independent of the origin of the tumour. Patients with an unknown status of prior SSA therapy or a SSA treatment longer than 28 days ago were excluded prior to analysis. The cohort consisted of patients with and without prior SSA treatment, which was determined by the treating endocrinologist/oncologist in line with the interdisciplinary tumour board, completely independent from the imaging procedure. The study was performed in compliance with the principles of the Declaration of Helsinki and its subsequent amendments (21), and with the approval of the local ethics committee (approval number 21-0102).

### 2.2 PET/CT imaging

SiTATE was obtained from ABX, Advanced Biomedical Compounds (Radeberg, Germany) and [^18^F]SiTATE was synthesized as described previously (17, 18, 22). All quality control data met the release criteria. [^18^F]SiTATE-PET/CT scans were acquired at the Department of Nuclear Medicine, LMU Munich on a Siemens Biograph mCT flow (Siemens Healthineers, Erlangen, Germany). After intravenous injection of 3 ± 1 MBq/kgBw (median 232 ± 36 MBq, range 152 to 310) of [^18^F]SiTATE, PET scans were acquired 90 ± 15 min after injection for 15-20 min (in flow mode depending on the body height). Patients were asked to empty the bladder if necessary. In 75/77 patients, contrast-enhanced CT scans with 1.5 mL of iopromide (Ultravist 300, Bayer Healthcare, Leverkusen, Germany) per kilogram of body weight were performed for anatomic localisation; the remaining two cases received diagnostic CT scan without contrast enhancement. The PET scan was acquired by static emission data with a scan speed of 0.7 mm/s for both neck and abdominal region and 0.9 mm/s for the lung region in flow mode. With CT scans serving for attenuation correction, PET images were reconstructed with a transaxial 200 × 200 matrix using TrueX (including TOF, 2 iterations and 21 subsets, 3D Gauss post-filter of 4-mm full-width-half-maximum).

### 2.3 Image analysis

Image analysis was performed using a dedicated software package (Hermes Hybrid Viewer, Hermes Medical Solutions, Stockholm, Sweden). Uptake in normal organs and tumour uptake (hottest lesion for each metastatic tissue type) in patients was assessed by SUV_max_ and SUV_mean_ (threshold 50% of max) measurements as described previously (18, 20). In short, spherical VOIs were placed inside the organ parenchyma using a 1-cm diameter VOI for small organs (adrenal glands) and a 2-cm diameter VOI for muscle, liver, spleen, fat tissue, aortic lumen (descending aorta), lung, bone (femur) and small intestine. Tumour-to-liver ratios (TLR) and tumour-to-background ratios (corresponding background for each lesion type, e.g. bone for osseous metastasis) were calculated for all measured tumour lesions according to the clinically relevant Krenning score which has been evaluated for SSTR-PET imaging (23, 24).

### 2.4 Statistical analysis

Data are reported as mean or median ± standard deviation or range as stated. Demographics and radiotracer uptake of normal organs (spleen, adrenal gland, liver small intestine, blood-pool, lung, bone) and tumour lesions were compared between group using a student’s t-test for metric variables and a Fisher exact/Chi-square test for contingency analysis of non-metric data. To compare the tumor uptake between SSA+ and SSA- patients, a lesion-based approach was used where the hottest lesion from each patient (if applicable) was included for different metastatic sites. Radiotracer uptake was correlated with the time after SSA-injection using a Pearson’s correlation coefficient. GraphPad Prism (version 8.4.3, GraphPad Software Inc., San Diego, CA) was used for statistical analysis and illustration of results. A significance level of p < 0.05 was applied in all analyses.

## 3 Results

### 3.1 Patients

All patients tolerated the examination well and did not report any unforeseen symptoms or adverse reactions. No drug-related pharmacologic effects or physiologic responses occurred. Thirty-seven male and forty female patients (SSA+ ♂22 ♀18; SSA- ♂15 ♀22) with differentiated NETs and a median age of 63 years (range 24 – 86 years) underwent a [^18^F]SiTATE-PET/CT. The mean age was comparable between groups and the time since initial diagnosis was significantly longer in the SSA+ group. Primary tumour locations included the small intestine (n=35), pancreas (n = 28), other gastrointestinal locations (n=5) and the primary tumour was not detectable (carcinoma of unknown primary) in n = 9 patients. Prior to PET/CT, the majority of patients were known to have hepatic (n = 51) metastases. Further metastatic sites included lymph nodes (n = 34), bone (n = 22), lung (n = 2) or peritoneal (n = 12) lesions. Most of the patients underwent surgery before (n = 45) followed by PRRT (n = 25) and chemotherapy (n = 14). In the SSA+ patients, the majority of primary tumours were located in the small intestine, whereas most of the patients in the SSA- group showed a pancreatic tumour. Also, more patients in the SSA+ group underwent surgery when compared to the SSA- and more SSA- patients received chemotherapy within their medical history compared to SSA+ patients. In SSA+ patients, the time interval between SSA treatment and PET/CT imaging was 12.9 ± 6.2 days. All SSA+ patients received the highest dose of the given SSA analogue (n=20 patients Sandostatin^®^ LAR^®^ 30 mg, n=20 patients Somatuline Autogel^®^ 120 mg). Detailed patient characteristics are provided in **Table 1**.

**Table 1:** Patient characteristics.

	All	SSA+	SSA-	p-value (SSA+ vs. SSA-)
Sex	♂37 ♀40	♂22 ♀18	♂15 ♀22	0.256
Age [y] (mean ± SD)	62.7 ± 12.1	63.7 ± 10.2	61.6 ± 13.9	0.455
Time since initial diagnosis [mo] (mean ± SD)	62.3 ± 57.6	75.3 ± 49.5	48.3 ± 62.9	0.039
Ki-67 (mean % ± SD) n=70	6.1 ± 5.3	5.1 ± 4.8	7.1 ± 5.7	0.127
Tumour-grading (G1/G2/G3) n=73	(25/47/1)	(16/23/0)	(9/24/1)	0.266
SiTATE dosage [MBq] (mean ± SD)	233 ± 36	240 ± 38	226 ± 32	0.069
Creatinine [mg/dl] (mean ± SD)	0.94 ± 0.26	0.92 ± 0.20	0.96 ± 0.32	0.612
Localization of primary tumour				
Pancreas/Small intestine/Other/CUP	28/35/5/9	7/29/2/2	21/6/3/7	<0.001
Prior therapies				
Surgery	45	31	14	<0.001
Chemotherapy	14	2	12	0.003
PRRT	25	18	7	0.007
Everolimus	2	0	2	
SIRT	10	6	4	
Radiotherapy	2	1	1	
Denosumab	1	1	0	

SSA, somatostatin analogue; ♂, male; ♀, female; MBq, Megabecquerel.

### 3.2 Biodistribution

**Figure 1:**
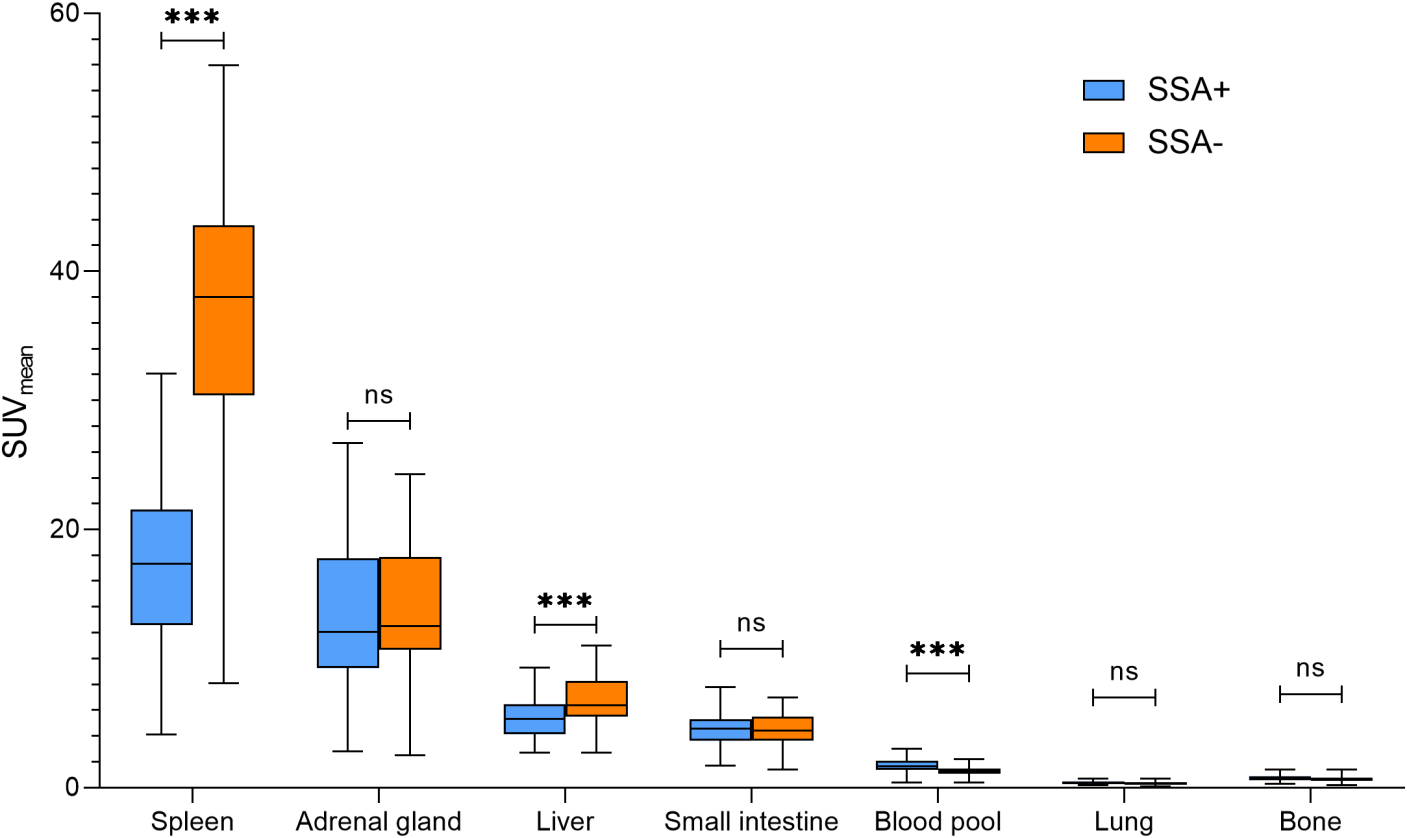
Biodistribution of [^18^F]SiTATE in normal organs in patients with (left boxplot)/without (corresponding right boxplot) SSA treatment. SUV, standardized uptake value; SSA, somatostatin analogue. *** p < 0.001, ns = p-value non-significant.

In line with previous studies, the radiotracer uptake in the normal organs was highest in the spleen, followed by the adrenal glands and the liver. Patients undergoing a SSA treatment showed a significantly reduced radiotracer uptake in the spleen (SUV_mean_ 17.5 vs. 36.7, p < 0.001) and the liver (SUV_mean_ 5.4 vs. 6.8, p < 0.001) when compared to patients without SSA treatment. On the other hand, the radiotracer uptake was significantly higher in the blood pool of patients with ongoing SSA treatment (SUV_mean_ 1.7 vs. 1.3, p < 0.001). For details of the biodistribution in normal organs see **Table 2** and **Figure 1**.

**Table 2:** Biodistribution of [^18^F]SiTATE in normal organs.

SUV_mean_ (mean ± SD)	All	SSA+	SSA-	p-value (SSA+ vs. SSA-)
Spleen	26.8 ± 12.9	17.5 ± 6.8	36.7 ± 10.3	< 0.001
Adrenal gland	13.5 ± 5.2	13.2 ± 5.7	13.7 ± 4.7	0.700
Liver	6.1 ± 1.8	5.4 ± 1.5	6.8 ± 1.8	< 0.001
Small intestine	4.6 ± 1.3	4.6 ± 1.4	4.6 ± 1.3	0.991
Blood pool	1.5 ± 0.5	1.7 ± 0.6	1.3 ± 0.3	< 0.001
Lung	0.4 ± 0.1	0.4 ± 0.1	0.3 ± 0.1	0.098
Bone	0.7 ± 0.2	0.7 ± 0.2	0.7 ± 0.2	0.400

SUV, standardized uptake value.

### 3.3 Tumour uptake and tumour-to-background ratios

**Figure 2:**
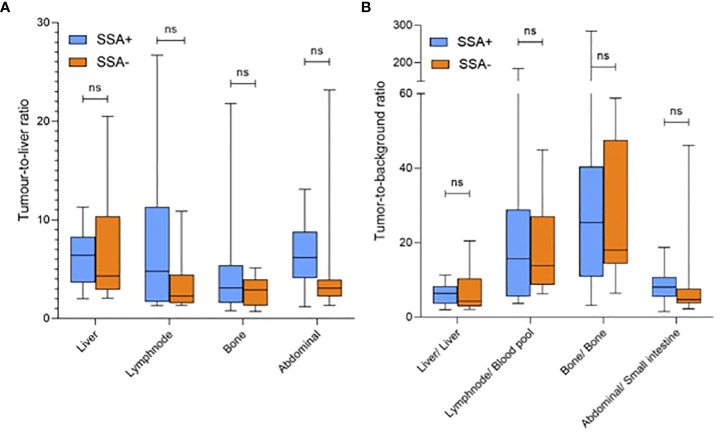
Radiotracer uptake of [^18^F]SiTATE in metastatic tumour lesions displayed as **(A)** tumour-to-liver and **(B)** tumour-to-specific background ratios (mean SUV_max_/SUV_mean_) for patients with (left boxplot)/without (corresponding right boxplot) SSA treatment. SUV, standardized uptake value; SSA, somatostatin analogue; ns = p-value non-significant.

Overall, the radiotracer uptake (SUV_max_) to primary or metastatic tumour lesions was not significantly different between patients with/without ongoing SSA treatment. Also, tumour-to-liver and tumour-to-specific-background ratios did not significantly differ between groups. For details of both groups see **Table 3** and **Figure 2**.

**Table 3:** Radiotracer uptake of [^18^F]SiTATE in metastatic tumour lesions.

	SSA+	SSA-	p-value (SSA+ vs. SSA-)
Tumour uptake (mean SUV_max_ ± SD)			
Hottest lesion (n=77)	43.5 ± 32.0; n=40	42.3 ± 36.9; n=37	0.969
Pancreas (n=33)	30.9 ± 46.0; n=7	43.8 ± 34.0; n=26	0.414
Bowel (n=8)	24.4 ± 15.7; n=6	15.4 ± 1.6; n=2	0.466
Lymph node (n=33)	35.8 ± 32.3; n=23	20.3 ± 13.2; n=10	0.154
Liver (n=50)	31.2 ± 14.5; n=29	39.3 ± 27.2; n=21	0.180
Lung (n=3)	16.5 ± 11.1		
Bone (n=26)	26.3 ± 40.2; n=15	15.1 ± 9.3; n=11	0.374
Heart (n=4)	12.1 ± 6.3; n=3	11.7; n=1	0.959
Soft tissue (n=1)	7.7		
Abdominal (n=23)	33.2 ± 16.3; n=13	35.8 ± 55.4; n=10	0.875
Spleen (n=1)	6.6		
Adrenal gland (n=1)	39.0		
Ovarium (n=1)		13.0	
Tumour-to-liver-ratio (mean SUV_max_/SUV_mean_ ± SD)			
Liver (n=50)	6.1 ± 2.8	7.1 ± 5.7	0.430
Lymph node (n=33)	7.1 ± 6.3	3.4 ± 2.9	0.094
Bone (n=26)	4.7 ± 5.1	2.7 ± 1.6	0.229
Abdominal (n=23)	6.4 ± 3.2	4.9 ± 6.5	0.477
Tumour-to-specific-background-ratio (mean SUV_max_/SUV_mean_ ± SD)			
Hepatic metastasis/Liver (n=50)	6.1 ± 2.8	7.1 ± 5.7	0.430
Lymph node metastasis/Blood pool (n=33)	28.1 ± 39.0	18.9 ± 14.0	0.477
Osseous metastasis/Bone (n=26)	40.5 ± 68.8	27.1 ± 18.4	0.538
Abdominal metastasis/small intestine (n=23)	8.6 ± 4.7	9.0 ± 13.2	0.917

SUV, standardized uptake value.

### 3.4 Individual radiotracer uptake under SSA treatment

**Figure 3:**
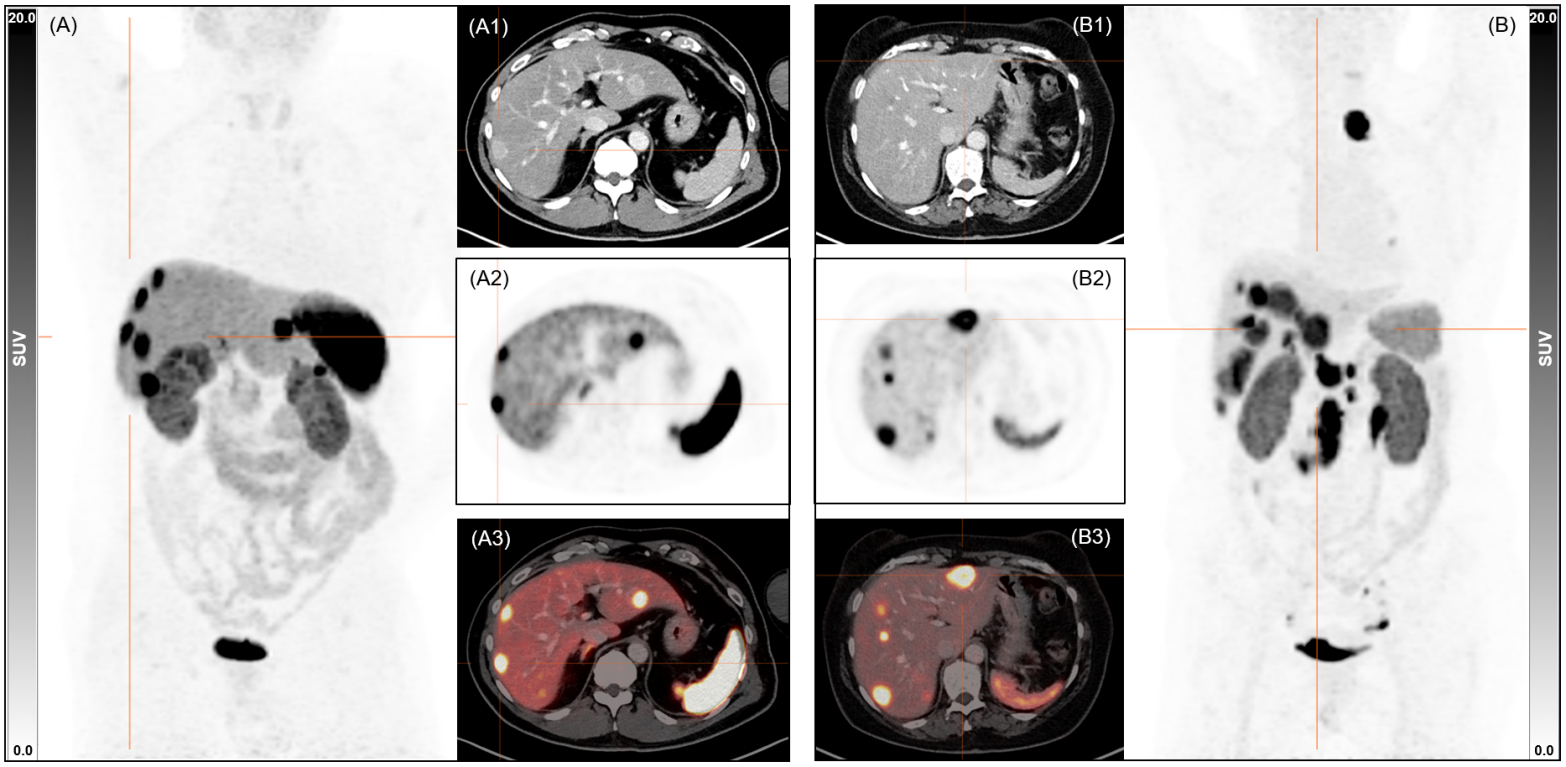
Exemplary maximum intensity projections images and axial sections (1 CT, 2 PET, 3 fused PET/CT) from patients **(A)** without SSA treatment (male, 59 y, 81 kg, 245 MBq, 89 min p.i., creatinine 1.0 mg/dl, liver SUV_mean_ 8.3, spleen SUV_mean_ 28.0, blood pool SUV_mean_ 1.3) and **(B)** undergoing SSA treatment (female, 71 y, 64 kg, 281 MBq, 93 min p.i., creatinine 0.7 mg/dl, liver SUV_mean_ 4.1, spleen SUV_mean_ 10.9, blood pool SUV_mean_ 1.6). SUV, standardized uptake value; SSA, somatostatin analogue; MBq, Megabecquerel.

Previous group-wise comparison suggests a reduced radiotracer uptake in normal organs but comparable tumour-to-background ratios. **Figure 3** shows two exemplary patient cases that visually match those results.

**Figure 4:**
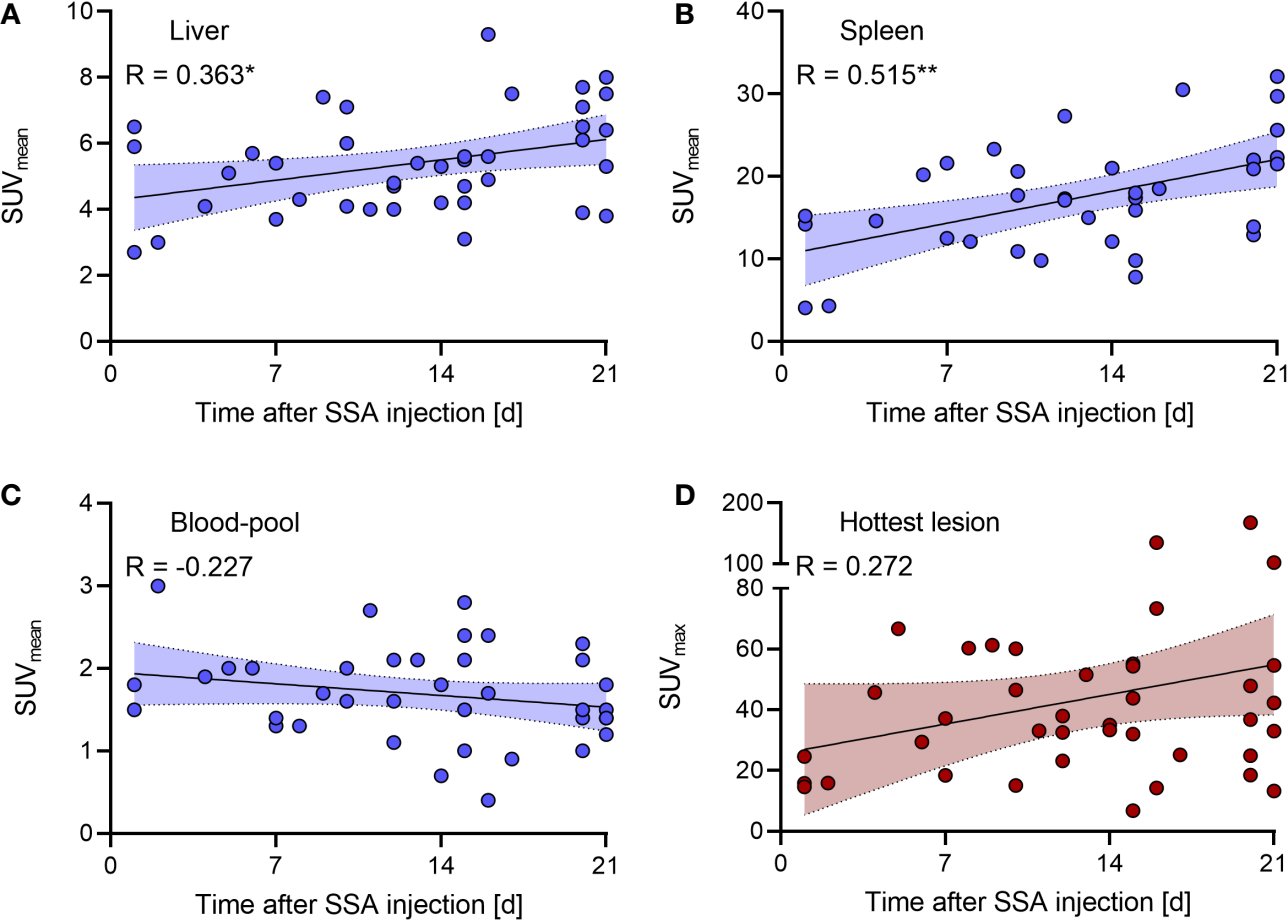
Inter-individual correlation of **(A–C)** SUV_mean_ of the liver/spleen/blood-pool and **(D)** SUV_max_ of the hottest lesion uptake with the time after SSA injection. The coloured fields around the correlation line represent the 95% confidence interval. SUV, standardized uptake value; SSA, somatostatin analogue. * and ** means a significant correlation.

Altered [^18^F]SiTATE uptake was time-dependent. **Figure 4** shows the inter-individual correlation between liver, spleen and blood-pool SUV_mean_ and hottest lesion SUV_max_ with the time after SSA injection with significant correlations for the liver and spleen radiotracer uptake (R_Liver_ = 0.363, p_Liver_ = 0.022; R_Spleen_ = 0.515, p_Spleen_ = 0.001).

## 4 Discussion

In the present study we investigated the influence of a previous treatment with long-acting non-radioactive SSAs on the SSR-expression in patients with GEP-NETs measured by PET/CT with the new radioactive SSA [^18^F]SiTATE.

Our results with [^18^F]SiTATE are in line with previous clinical studies with several SSR radioligands: Haug et al. investigated 105 NET patients with [^68^Ga]Ga-DOTATATE of whom 35 had been pre-treated with long-acting octreotide and reported a significantly lower [^68^Ga]Ga-DOTATATE uptake of non-tumorous spleen and liver in patients treated with SSAs (15). Lodge et al., who prospectively investigated the effect of a pre-dose of octreotide prior to injection of the radiotracer on the distribution of [^68^Ga]Ga-DOTATOC in 7 patients with GEP-NETs intra-individually within 7 ± 9 days observed a decreased radiotracer uptake in normal liver by 25% and spleen by 47% with octreotide pre-therapy (14). Another prospective study of Aalbersberg et al. intra-individually evaluated 34 patients with metastatic NET before and after lantreotide therapy with the result of significantly decreased uptake of non-tumorous liver and spleen (25). This is in accordance with our results with significant lower [^18^F]SiTATE background uptake (SUV_mean_) in liver and spleen tissue in patients with previous SSA treatment, compared to patients without.

Moreover, our results with [^18^F]SiTATE showed a significantly higher blood pool signal in SSA treated patients. This could reflect a decreased binding and therefore higher number of circulating radioligands because of two potential explanations: a lower SSR density as a consequence of predominantly internalized SSR 2 in neoplastic and physiologic target tissues or SSR saturation with non-radioactive SSAs under therapy (26).

In contrast to Haug et al., who used [^68^Ga]Ga-DOTATATE, in our study with [^18^F]SiTATE the radioligand uptake of the non-tumorous background in liver and spleen as well as in blood pool showed a time dependency on the interval since SSA treatment with significant positive correlations between the number of days after treatment and radiotracer uptake of liver and spleen, and a trend towards an inverse correlation with the blood pool. This time dependency on the interval since SSA treatment may be explained by the fact that the non-radioactive SSAs compete with the SSR radioligands for receptor binding sites and the quantity of SSR internalization is dependent on the octreotide dose (26). This means that the more non-radioactive SSAs are circulating in the blood, the more receptors are blocked and internalized, but with reversibility of the internalization over time (27).

Aalbersberg et al. observed an increased tumour uptake leading to an increased tumour-to-liver ratio (25). Former studies using conventional scintigraphy for the detection of NET metastases before and during treatment with octreotide showed an intra-individually improved visualization of carcinoid liver metastases in 5 midgut NET patients after SSA treatment measured by ^111^In-pentreotide (13) and a higher tumour-to-background ratio in 8 NET patients by using ^111^In-octreotide scintigraphy (28). In contrast, analogously to Haug et al. and Lodge et al. (14, 15), our results reveal no significant differences in SSR expression and target-to-background-ratios. With [^18^F]SiTATE there were no significant differences in SUV_max_ in tumours/metastastic lesions between the two groups as well as no significant differences in tumour-to-liver and tumour-to-specific-background ratios. Our findings may be partially explained by the fast and efficient internalization of SSR 2 after agonist stimulation under octreotide therapy *in vivo* in neoplastic as well as in physiologic SSR 2 target tissues (26, 27). Furthermore, an agonist-induced up-regulation of SSR subtypes, which causes an increase of the receptor density in the tumours and metastases of SSA treated patients (12) with a consecutively higher [^18^F]SiTATE uptake may be partially masked by SSA occupied receptors (15). Thus, there might be a steady state of concomitant receptor internalization and overexpression. In long-acting SSAs, the initial pharmacokinetic profile after injection differs between lanreotide depot and octreotide depot formulations (15, 29), while subsequent serum concentrations remainquite stable over 28 d with both formulations (15, 29).

It has to be mentioned that the above cited scintigraphical studies are limited by several aspects: first, only a very low number of patients was investigated. Secondly, the quantifiability in conventional scintigraphy is reduced compared to PET/CT with SUV calculations. Thirdly, interpretation of these intra-individual scintigraphy results is limited by a possible tumour progression during the 12 month treatment course which may result in an increase of the uptake values.

However, all these study results consistently indicate that octreotide treatment may influence the binding and change the biodistribution of SSR radioligands, but suggest that the diagnostic reliability of somatostatin receptor imaging in NET metastases is not significantly compromised by any previous or concomitant octreotide therapy (13). Moreover, these findings even underline that SSA treatment may facilitate the detection of NET metastases, mainly driven by a decline in background binding in the liver and spleen rather than an increase in tumour binding, possibly providing an improved tumour delineation (15).

Because of the heterogeneity of NETs and consequently the possibility of various biologic behaviours, it remains uncertain if these findings can be generalized to other types of NET (15). Also, the small sample size and heterogeneity of patients might impair the detection of statistically significant differences between groups. There are differences in contributions of tumor grading, metastatic sites or time since diagnosis that cannot be avoided due to the individual clinical courses of included patients in this rare disease. Consequently, one major limitation of this study lies in the inter-patient comparison of binding. In most of the patients, treatment with SSAs cannot be paused which excludes them from an intra-patient comparison. Nevertheless, the presented results are promising, but require further investigation in future clinical trials to validate our data intra-individually and also to evaluate the influence of long-acting SSA pre-treatment on radioligand binding in patients that receive PRRT with e.g. [^177^Lu]Lu-DOTATATE.

## 5 Conclusions

A treatment with long-acting SSAs does not reduce the [^18^F]SiTATE -binding in tumorous target lesions of GEP-NET patients and even reveals a significant lower background signal in non-tumorous liver and spleen tissues, consistently to other radioactive SSA, which could improve demarcation of metastases in these organs. Our results add support to the hypothesis that a previous or concomitant treatment with long-acting SSAs does not unfavourably/adversely influence the SSR expression and therefore confirm the clinical approach not to discontinue/interrupt any SSA medication prior to a PET/CT examination.

## Data availability statement

The raw data supporting the conclusions of this article will be made available by the authors, without undue reservation.

## Ethics statement

All procedures performed in studies involving human participants were in accordance with the ethical standards of the institutional and/or national research committee (LMU Munich - approval number 21-0102) and with the 1964 Helsinki declaration and its later amendments. The patients/participants provided their written informed consent to participate in this study.

## Author contributions

RE: data acquisition, statistics, writing of the draft. MH, LS, LU, GS, RT, MB, VW, FD, MZ, AT, HI, FG, CC, MU, JR, PB: data acquisition, revision of the manuscript. AD: image reconstruction, revision of the manuscript. SL, KJ, CW, BW, RS: SiTATE preparation, revision of the manuscript. TK, TP: histopathological correlations, revision of the manuscript. SB, CW, CS, CA: patient referral, clinical data acquisition, revision of the manuscript; LB: concept, data acquisition, statistics, revision of the manuscript. All authors contributed to the article and approved the submitted version.
